# Editorial: Sport practice and physical activity—the social function of sport in contemporary societies

**DOI:** 10.3389/fspor.2025.1693014

**Published:** 2025-09-19

**Authors:** Pedro Moreira Gregori, Juan Carlos Martin

**Affiliations:** Institute of Tourism and Sustainable Economic Development, University of Las Palmas de Gran Canaria, Las Palmas de Gran Canaria, Spain

**Keywords:** sport practice, physical activity, social function of sport, contemporary societies, theoretical-practical approaches

**Editorial on the Research Topic**
Sport practice and physical activity—the social function of sport in contemporary societies

Sport and physical activity are essential for developing healthy, dynamic, and cohesive societies. Besides its recreational dimension, sport has a significant impact on aspects such as health, education, social inclusion, and local development. That is why various public policies promote greater participation and equitable access to facilitate the practice of sports and physical activities.

The thematic focus of this special issue is to analyse the complex interactions between sport, physical activity, active living, mobility, and the factors that influence the adoption of an active lifestyle. This integrative perspective is essential to understand better how social structures, urban design, individual motivation, and public policies may or may not facilitate the adoption of active habits.

Understanding the main drivers and barriers that motivate or demotivate physical activity, also considering the intrinsic complexities of each of the societies analysed in the case studies presented, is crucial to designing better policies that foster physical activity. By transferring theoretical advances into practical strategies that promote healthier and more active lifestyles, these heterogeneous and pluralistic approaches could contribute to the development of more targeted policies and interventions adapted to different population groups (according to age, gender, socioeconomic status, and socio-cultural context).

Special consideration has been given to the multidimensional impact of sport and physical activity, with particular emphasis on their socio-economic influence, their innovative potential, and their role in promoting health, inclusion, and cultural identity. Ethical issues, psychosocial effects on well-being, and the relationship between sport and urban development, as well as physical barriers, have also been considered.

The 11 articles that we present belong to a diverse group of 41 researchers from universities and research centres in Australia, Belgium, China, Finland, Germany, the Netherlands, Norway, Spain, Turkey, the United Kingdom and the United States. We provide a summary of them below in order from newest to oldest based on their publication dates.

Physical activity in the EU was analysed by Martín and Moreira using data from the latest Eurobarometer on sport and physical activity. They applied a hybrid fuzzy analysis approach to calculate a synthetic index measuring the physical activity of EU citizens. The method was applied to the entire dataset obtained from 26,578 respondents. The results revealed, among other findings, territorial inequalities in Europe, mainly between north and south and, to a minor extent, between east and west. Higher rates of exercise and physical activity in northern countries could be attributed to the development of infrastructure for non-motorised mobility. Southern and some eastern European countries are more sedentary, mainly due to cultural and climatic factors, as well as a possible lack of integrated strategies to promote physical activity. They also analysed the influence of other covariates that affect the latent variable under study, such as age, social class, educational level, gender, life satisfaction and positive perceptions of the EU. All of which provides interesting information for the potential development of specific programmes to reduce a sedentary lifestyle.

Koç et al. studied the relationship between perceived freedom in leisure time and multifaceted leadership orientations among 633 residents of Turkish provinces who participate in recreational sports in nature. They concluded that participants demonstrate high levels of perceived freedom in leisure time and moderate levels of leadership orientation. Analysis of several variables revealed a strong positive relationship between levels of perceived freedom in leisure and multifaceted leadership orientations, which would derive from the willingness to prioritise one's own desires, emotions and thoughts to feel free during leisure time. It also highlights that there are no significant differences according to age, educational level, and gender, confirming the findings of other studies on gender equality in open-air sports. However, higher scores in political leadership were observed in men. In relation to marital status, they conclude that single people have a higher perception of freedom in leisure than married people. Other variables that influence the perception of freedom and leadership characteristics are the type of discipline, its duration, and the level of personal well-being.

Currently, only around half of US citizens achieve the recommended physical activity patterns, according to Martin et al. In their article, they highlight that personality characteristics, encompassed by determination and resilience, should be beneficial in overcoming common barriers that inhibit participation in physical activities. Authors performed a literature review aligned with the guidelines for preferred data sets for systematic reviews and meta-analyses (PRISMA), focusing on 33 studies of 37,370 respondents of diverse ages, genders, and cultures. Most studies found positive relationships between determination, resilience, and physical activity outputs, such as adherence, intensity, and performance in competitive environments. Results indicated that the personality characteristics of determination and resilience play an essential role in supporting participation in physical activity. And that individuals who show higher resilience are more likely to engage in physical activity more regularly and achieve better performance results, highlighting the association between tenacity and resilience with participation and adherence towards physical activity.

Meanwhile, Chen et al. presented a study on leisure restrictions and the negotiation of structural relationships among recreational divers. They indicated that diving has become a very popular recreational activity in China over the last two decades. The study combined in-depth interviews with 20 diving enthusiasts with the Positive and Negative Affect Schedule (PANAS). The study found that diving enthusiasts predominantly use cognitive strategies to address personal and interpersonal constraints, while behavioural strategies are more effective on structural constraints. This supports the theoretical perspective on the relationship between types of constraints and negotiation strategies. Compared to Western contexts, family dynamics in China have a more pronounced influence on the configuration of interpersonal constraints, as well as economic constraints and more limited access to resources.

Indelicato studies women's participation in sport, using data from Special Eurobarometer 525. Using the Fuzzy-Hybrid approach and multinomial logistic regression, he analysed the influence of gender, age, income, education, political beliefs, and country of residence on such participation. The results highlight some geographical differences: Nordic countries, headed by Sweden and Finland, have more favourable scores in terms of opinions on women's equality of participation in sport. Meanwhile, Austria and Eastern European countries maintain a more traditional and conservative view of gender roles. More favourable opinions are also expressed by those with leftist beliefs and the highest degree of life satisfaction.

The impact of social exclusion on participation in experiential sports was analysed by Li and Qu. Based on the theory of social exclusion, they developed a conceptual model in which social exclusion is the independent variable, the intention to participate in experiential sports is the dependent variable, and loneliness and the need for social connection are the mediating variables. Using an online survey of 415 respondents in Shanxi Province (China), they analysed the data using structural equation models and the Bootstrap method. Results indicated that social exclusion significantly and positively predicts the intention to participate in experiential sports consumption. It also has a significant positive effect on loneliness and the need for social connection. However, loneliness does not mediate the relationship between social exclusion and the intention to consume experiential sports, while the need for social connection does.

Spruijtenburg et al. studied the role of teammates, coaches, and support in adolescent sports participation. In the study, they analysed the associations between different social actors (family, teammates, coaches, professors) and types of support (emotional, instrumental, co-participation) and the hours of participation in organised sports among secondary school students in the USA (*N* = 294). Using multilevel fixed-effects linear regression models, they found significant associations between various social actors and types of support and participation in organised sports. Support from teammates and coaches, as well as instrumental support, emerged as the strongest predictors of participation. Furthermore, they observed that these associations remained stable over time. However, they also observed substantial individual variability in the relationship between social support and participation.

Regarding the impact of large-scale sporting events on national identity, Wang et al. analysed the profound effects on improving residents’ national identity. They took the 2023 Asian Games in Hangzhou, China, as a case study. They analysed the responses of 1,096 residents to a survey, evaluating their interrelationships using structural equation modelling and bootstrapping. The results highlighted that these Games had a significant positive impact on national identity, subjective well-being, and city image. They concluded that the degree of participation in large-scale sporting events has a direct positive effect on national identity, and that subjective well-being and city image partially mediate this effect.

Morgan et al. present an exploratory qualitative study on the experiences of women in non-sporting volunteer roles in Australian community football clubs. The aim was to understand the barriers and facilitators to such participation. Semi-structured interviews were conducted with six women from four clubs. The analysis of barriers highlighted high self-expectations, limited resources, organisational structure, ethnicity, maternity, and gender roles. Facilitators included self-confidence, social connection, and institutional support. The results also revealed the considerable joint interaction of gender, ethnicity, maternity, and organisational and socio-cultural variables.

According to Biz and Schubert, the participation of famous athletes in supporting social causes has become a frequent practice in recent years. Based on 12 semi-structured interviews with active professional athletes from different continents and recently retired, the authors explore the most remarkable attributes of their personal brands. They conclude that, in contrast to the commercial characteristics of products, the attributes of athletes’ personal brands are considered more critical for the achievement of social causes. Attributes in the field of sport, such as professional achievements and behaviour during competitions, are considered the primary sources of credibility. Consistency and alignment between the profiles of sponsors and social causes are perceived as relevant and determining factors in the success of sponsorship activities.

Gil-Beltrán et al. analysed how physical activity in the company of others and the prioritisation of positivity contribute to it becoming a habit, through the upward spiral theory of lifestyle change. They evaluated the impact on the relational loop of physical activity, emotions, and commitment. Using structural equation modelling and multilevel analysis, they analysed two studies in Spain (*N* = 553 and *N* = 146, participants who exercised regularly). The results indicated that people exercise more frequently when they experience higher levels of commitment and positive emotions, especially when they exercise with other people, and prioritise positivity. Furthermore, positive emotions during physical exercise enhance the relational cycle between emotions and exercise.

